# One-step synthesis of carbon-supported electrocatalysts

**DOI:** 10.3762/bjnano.11.126

**Published:** 2020-09-17

**Authors:** Sebastian Tigges, Nicolas Wöhrl, Ivan Radev, Ulrich Hagemann, Markus Heidelmann, Thai Binh Nguyen, Stanislav Gorelkov, Stephan Schulz, Axel Lorke

**Affiliations:** 1 Faculty of Physics and CENIDE, University of Duisburg-Essen, Carl-Benz-Straße 199, 47057 Duisburg, Germany; 2 The hydrogen and fuel cell center (ZBT GmbH), Carl-Benz-Straße 201, 47057 Duisburg, Germany; 3 Interdisciplinary Center for Analytics on the Nanoscale, University of Duisburg-Essen, Lotharstraße 1, 47057 Duisburg, Germany,; 4 Faculty of Chemistry and CENIDE, University of Duisburg-Essen, Universitätstraße. 5-7, 45141 Essen, Germany

**Keywords:** electrocatalyst, fuel cells, hybrid nanomaterial, long-term stability, nanoparticle embedding, one-step synthesis, plasma-enhanced chemical vapor deposition (PE-CVD)

## Abstract

Cost-efficiency, durability, and reliability of catalysts, as well as their operational lifetime, are the main challenges in chemical energy conversion. Here, we present a novel, one-step approach for the synthesis of Pt/C hybrid material by plasma-enhanced chemical vapor deposition (PE-CVD). The platinum loading, degree of oxidation, and the very narrow particle size distribution are precisely adjusted in the Pt/C hybrid material due to the simultaneous deposition of platinum and carbon during the process. The as-synthesized Pt/C hybrid materials are promising electrocatalysts for use in fuel cell applications as they show significantly improved electrochemical long-term stability compared to the industrial standard HiSPEC 4000. The PE-CVD process is furthermore expected to be extendable to the general deposition of metal-containing carbon materials from other commercially available metal acetylacetonate precursors.

## Introduction

The global fuel cell market reached a value of $4.5 billion USD in 2018 and is projected to reach a value of $9.5 billion USD by 2024, resulting in a compound annual growth rate of 13.2% during 2019–2024 [1]. Especially in the transport industry, fuel cells are expected to play a significant economic and ecological role when it comes to environmentally friendly energy production due to their ability to directly convert the chemical energy of a variety of fuels (including those produced by renewable energy) to electricity at high rate and efficiency, while mostly producing ecologically friendly by-products [2–3].

Unfortunately, there are still many scientific challenges regarding the commercial production of fuel cell catalysts, especially the scarcity of noble metals and the insufficient electrochemical long-term stability. Even though the surface-to-volume ratio can be drastically increased by the use of nanoparticles instead of thin films, the amount of noble metal (usually platinum group metals) required for electrode materials produced by conventional synthesis approaches is still cost-inefficient for broader commercial application [4–5]. Furthermore, since surfactants (capping agents) are typically applied in the traditional wet chemical synthesis of metal nanoparticles in order to control their morphology and size, additional surface activation steps are necessary to remove these organic molecules from the particle surface (reactivation) [6–8], which often further increases production costs. A similar issue limits surfactant-free chemical synthesis techniques, such as solvothermal and hydrothermal synthesis [9]. In these processes, long ultrasonication as well as reduction/heating procedures are required [6,10], both of which will impede industrial production. In contrast, physical approaches for the synthesis of electrocatalysts (such as plasma-assisted techniques [11] and laser ablation [12]) are surfactant-free and scalable. However, these approaches typically require multiple-step procedures in which the support and catalytic nanoparticles (NPs) are first produced individually and then combined in a third step (i.e., NP sedimentation in liquid phase [13] or the supercritical CO_2_ method [14]) to form the required electrocatalyst [6]. The third step is technically challenging and has a substantial influence on the homogeneous distribution of NPs on the support. Since the NPs are typically bound to the support by weak van der Waals forces, the electrochemical long-term stability of these electrodes is often unsatisfactory due to particle coalescence and Ostwald ripening [15]. These degradation processes can be avoided by increasing interparticle distance (at the expense of the electrochemically active surface) or by creating barriers to prevent NP agglomeration (i.e., highly porous supports) [16].

After years of development, conventional synthesis methods still have problems meeting the requirements for scalability of the synthesis and long-term stability of the resulting catalyst. In this work, we report on a novel one-step synthesis approach that not only addresses the above-discussed challenges but also represents a reproducible method, which in principle is scalable, for the production of carbon-supported electrocatalysts developed on the basis of a previously reported process [17]. An inductively coupled plasma-enhanced chemical vapor deposition process at a substrate temperature as low as 350 °C using platinum acetylacetonate as a single-source precursor was established for the deposition of a Pt/C electrocatalyst. Platinum in the form of NPs is homogeneously distributed in a carbon support structure due to the simultaneous deposition of both the C-matrix and the Pt-NPs during the process. The carbon support consists of carbon nanowalls (CNWs), which are vertically aligned, multilayer graphene nanosheets [18–19]. The morphology of the CNWs, the amount of platinum loading as well as the size of the platinum NPs can be precisely adjusted, and Pt-NPs with a mean particle diameter less than 3 nm and a narrow particle size distribution (PSD) with a geometric standard deviation of 1.24–1.3 can be achieved. Furthermore, the NP immobilization within the carbon support significantly improves the long-term stability of the catalyst, as shown by cyclic voltammetry (CV) measurements.

## Results and Discussion

### One-step synthesis and its product

**Figure 1:**
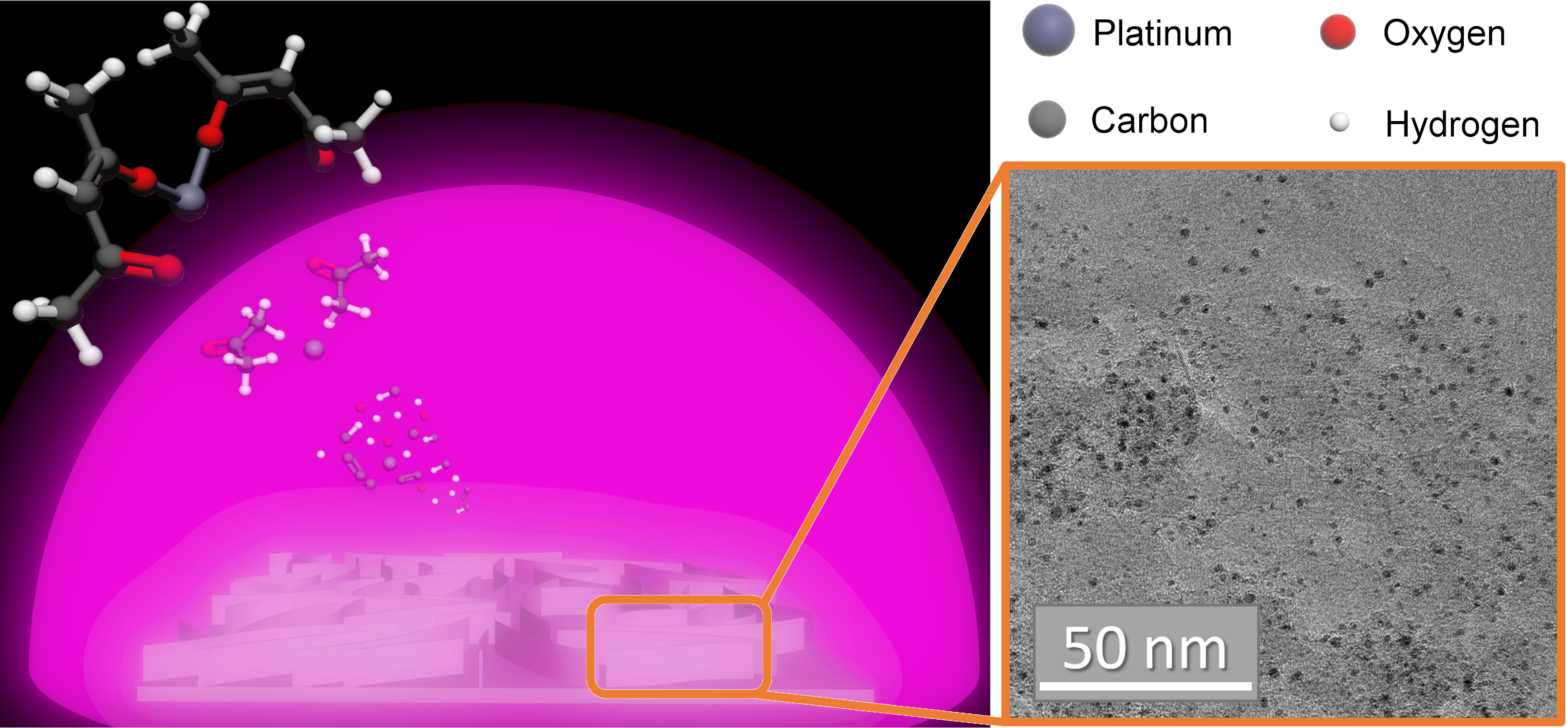
Schematic illustration of the deposition process. Platinum acetylacetonate is dissociated by the plasma and the simultaneous deposition of Pt-NPs (black dots in the TEM image) and CNWs occurs.

Platinum acetylacetonate was used as a single-source precursor for the deposition of Pt-NPs embedded in the supporting CNW matrix. CNWs are vertically aligned, graphitic, multilayer carbon sheets, which provide not only a high surface area but also a decent electrical conductance, thus acting as an ideal support material for electrocatalysis [18–19]. The route for the simultaneous growth of both the Pt precipitates and the carbon matrix (CNWs), which merge to form the catalytic hybrid material, is illustrated in Figure 1. For details on the experimental procedures, see the Experimental section.

**Figure 2:**
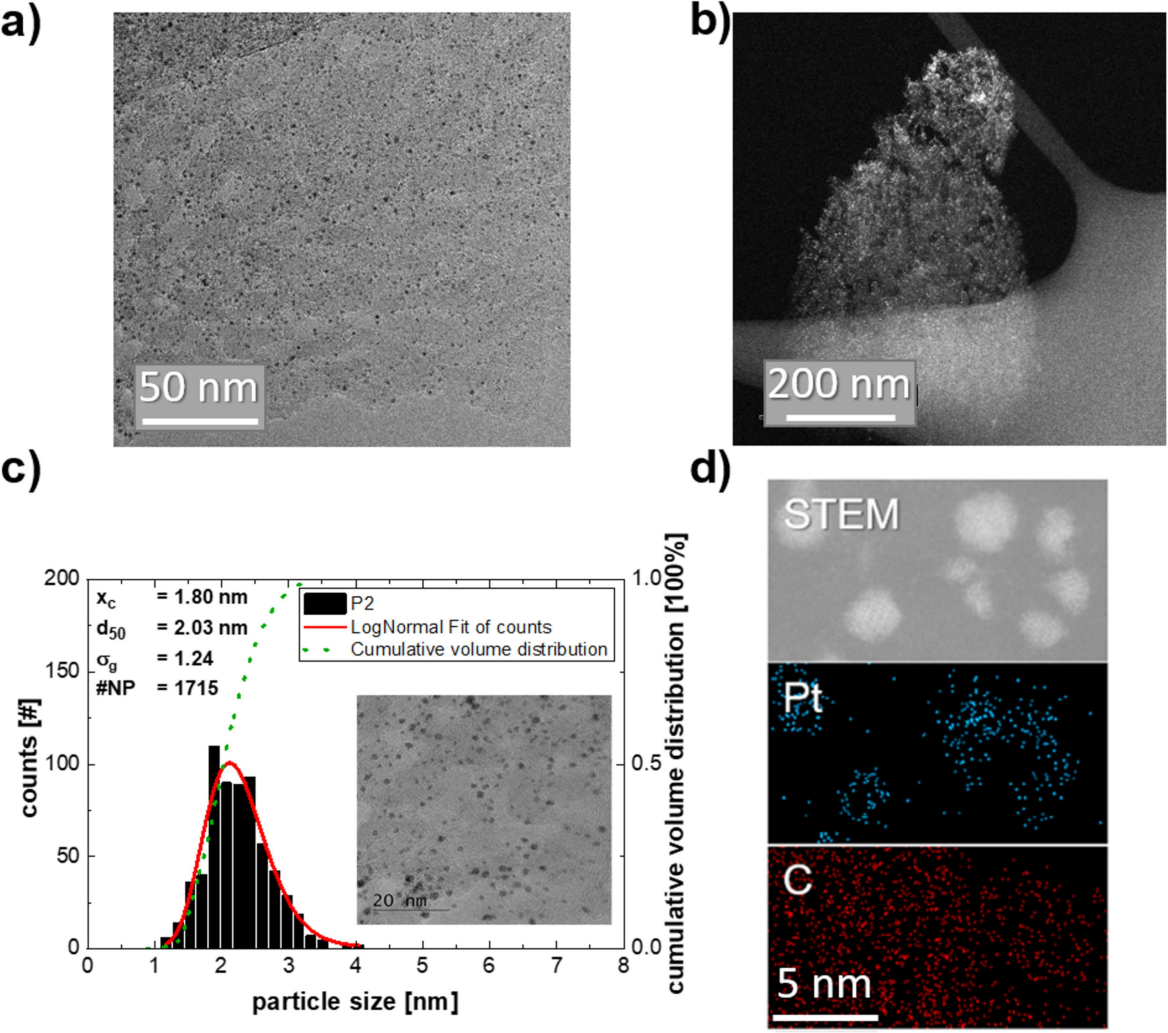
a) Bright-field TEM micrograph of a typical Pt/CNW sheet, showing homogeneously distributed Pt-NPs (black dots). b) A dark-field TEM micrograph of a porous CNW. c) Histogram of the PSD of Pt-NPs, proving a narrow PSD with a geometric standard deviation of 1.24 and a small mean particle diameter of 1.8 nm. d) Dark-field STEM micrograph and respective EDS measurements of the Pt and C distribution of a small area of a Pt/CNW sheet. The black dots in a) are identified as Pt-NPs. No other elements than Pt, C, and O were detected, which was later confirmed by XPS measurements (see Figure 5 a).

A typical CNW sheet of a sample processed at 8 Pa chamber pressure, 60 sccm argon carrier gas flow rate, and 350 °C substrate temperature is shown in a bright-field transmission electron microscope (TEM) micrograph in Figure 2a. Pt-NPs (black dots) are homogeneously distributed across the entire porous carbon sheet (grey areas). The porosity of the support can be observed in the dark-field TEM micrograph (Figure 2b) and has been reported to be beneficial in electrocatalysis, as it reduces mass transport limitations and increases the surface area [20]. A typical histogram of the Pt-NPs is shown in Figure 2c, displaying a narrow PSD with a geometric standard deviation [21] between 1.24 and 1.3. Such a narrow PSD, in combination with a small particle size, is essential to reach high mass activities in catalysis [22–23]. Figure 2d shows a dark-field scanning transmission electron micrograph (STEM) of several Pt-NPs on a CNW sheet and corresponding energy dispersive spectroscopy (EDS) measurements of the platinum and carbon content. No significant amounts of other elements were detected. These measurements confirm that the black dots in the bright field micrographs are indeed Pt-NPs.

**Figure 3:**
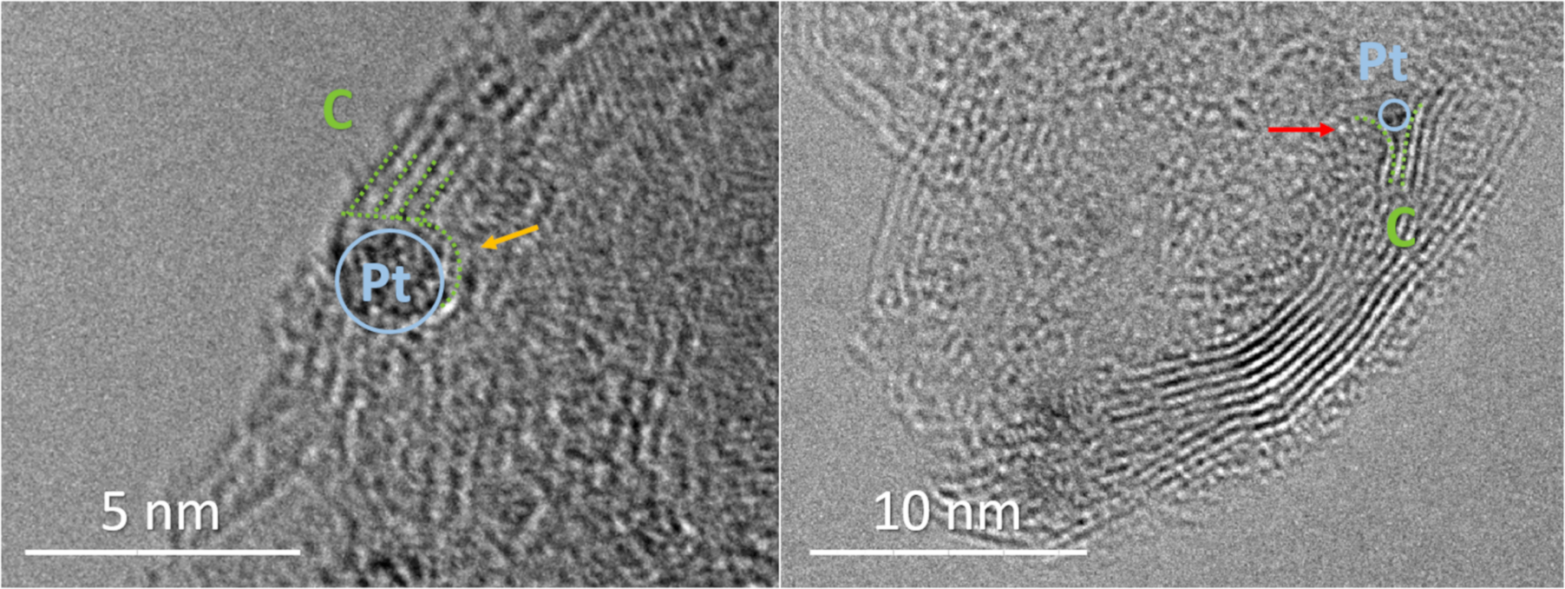
TEM micrographs of Pt-NPs (blue) at the edge of a CNW sheet. The carbon layers (green) are disturbed (bent/split) by the Pt-NPs (see yellow and red arrow).

The co-deposition of platinum and carbon during the PE-CVD process guarantees a homogeneous distribution of the Pt-NPs in the C matrix. Even though the exact position of the Pt-NPs is difficult to determine by projective imaging (such as TEM), ultrahigh resolution TEM images prove the formation of embedded Pt-NPs in the carbon matrix by resolving individual carbon layers, which are affected by the Pt-NP. In the left frame of Figure 3, it can be seen that the Pt-NP (blue) interrupts/disturbs some of the carbon layers (green), whereas one of the layers appears to bend around the Pt-NP (yellow arrow). In the right frame, the carbon layers split up as they pass around the Pt-NP (red arrow). Both of these phenomena and the improved electrochemical stability (see section “Electrochemically active surface area and long-term stability”) support the assumption that the Pt-NPs are embedded in the carbon matrix.

The proportion of carbon etchant (H and O) to CNW growth species (CH and C_2_) in the plasma, which is frequently regarded as one of the most critical parameters of crystalline CNW growth [19,24–27], is well-adjusted under the given deposition conditions and, as a result, promising electrocatalysts are obtained. However, in order to assess how performance-critical properties such as CNW morphology, Pt-loading, and PSD of the resulting Pt/CNW catalysts can be influenced by specific process parameters, these details were systematically studied. A table summarizing the process parameters for every sample shown in this work can be found in Supporting Information File 1, Table S1. It is worth noting that under identical synthesis parameters, the results are reproducible.

### Controlling the physical properties of the support

**Figure 4:**
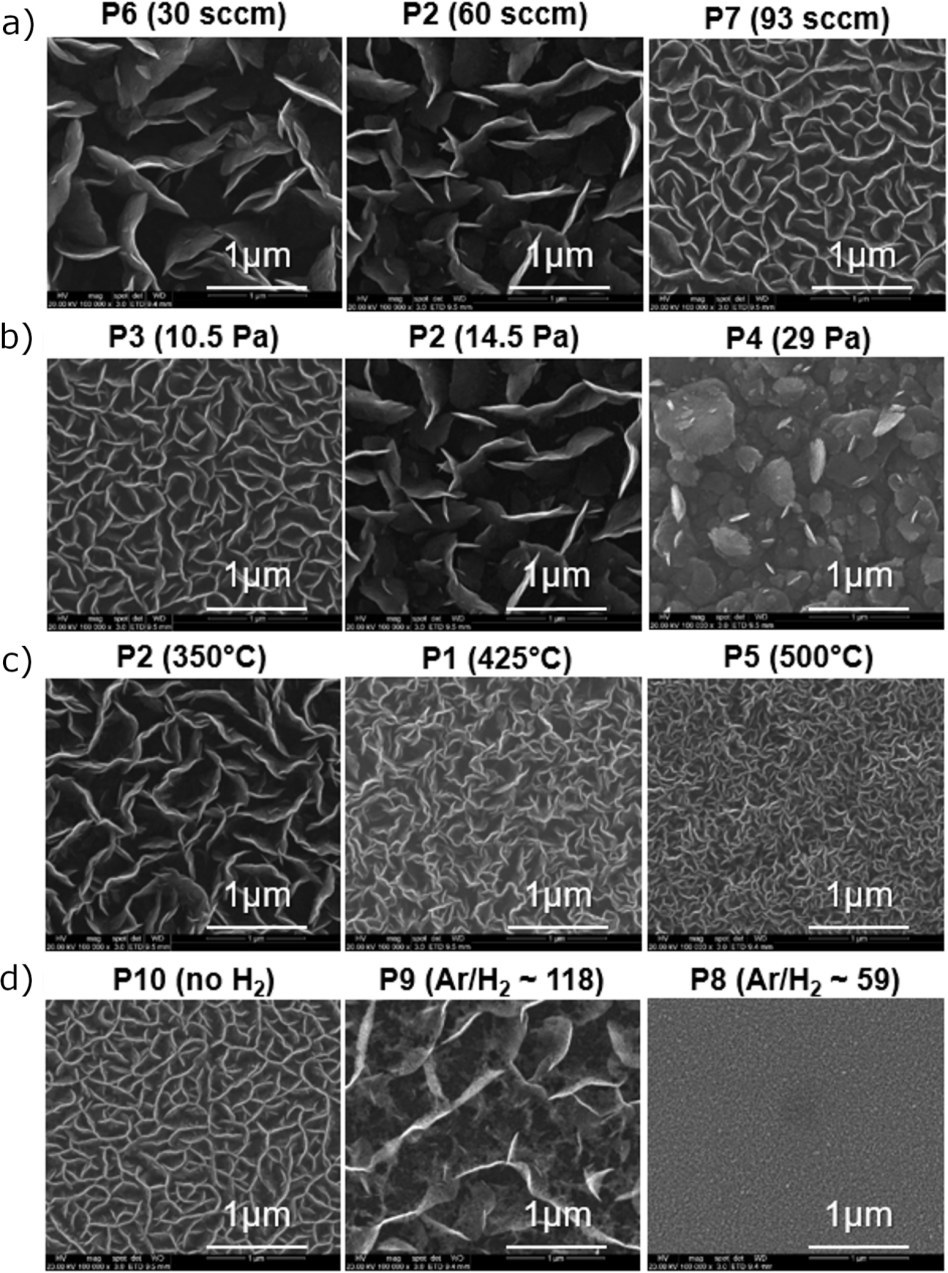
SEM images of selected CNW morphologies of samples processed at varying process parameters: a) argon carrier gas flow rate, b) process pressure, c) substrate temperature, and d) hydrogen addition. Only one parameter was varied at a time, while the pressure was kept constant at 14.5 Pa for a) and c) and at 10.5 Pa for d), the carrier gas flow rate was kept constant at 60 sccm for b–d), and the temperature was kept constant at 350 °C for a), b) and d). All four process parameters influence the density and thickness of the CNWs. High pressures and low carrier gas flow rates enable a morphology transition from CNWs to nanoflakes.

Figure 4 shows the influence of the carrier gas flow rate (a), pressure (b), and substrate temperature (c) on the CNW morphology. The wall density, as well as the growth rate, was found to increase with increasing gas flow rate, decreasing pressure and increasing substrate temperature, which agrees with a previous study [17]. Figure 4d shows the influence of H_2_ content in the carrier gas on the CNW morphology. Increasing the H_2_ supply results in an increase of atomic H in the plasma and a decrease of the O content, whereas the CH and C_2_ signals remain stable, as was confirmed by optical emission spectroscopy (OES, see Supporting Information File 1, Figure S3). More remarkably, the density of the CNWs decreases drastically when increasing the hydrogen concentration in the plasma (Ar/H_2_ ≈118, Figure 4d, middle panel). Comparable results were previously reported by Cho et al. [28] and Suzuki and coworkers [29]. The reduction of the density of the CNWs results from the etching of the CNWs at the initial nucleation step by atomic hydrogen, effectively reducing the nucleation sites. Increasing the H_2_ concentration in the plasma finally results in the deposition of an amorphous layer (Ar/H_2_ ≈59, Figure 4d, right panel).

Raman spectroscopy was performed to determine the quality (defect density, defect type, and hybridization) of the deposited Pt/CNW layers. All samples produced at sufficiently high pressures and low carrier gas flow rates exhibit the typical spectrum observed for CNWs, with peaks resulting from their graphitic structure and their sharp, exposed edges [30]. At low pressures and high carrier gas flow rates, the intensity of the defect-induced peak D’ increases, as observed in a previous study [17]. The amorphization stage of all samples is 1, which indicates 100% sp²-hybridization [31]. No peaks are found for the Pt-NPs in the investigated wavenumber region, which is supported by a study of Kimata et al. [32]. A more detailed evaluation of the Raman data can be found in Supporting Information File 1.

### Controlling the physical properties of the active material

Since the electrocatalytic performance of the Pt/CNW hybrid material is expected to depend on the amount, size, shape, distribution and oxidation of the embedded Pt-NPs [4], we carefully studied the influence of the four process parameters presented above on the physical and chemical properties of the resulting Pt-NPs.

#### Platinum loading and degree of oxidation

**Figure 5:**
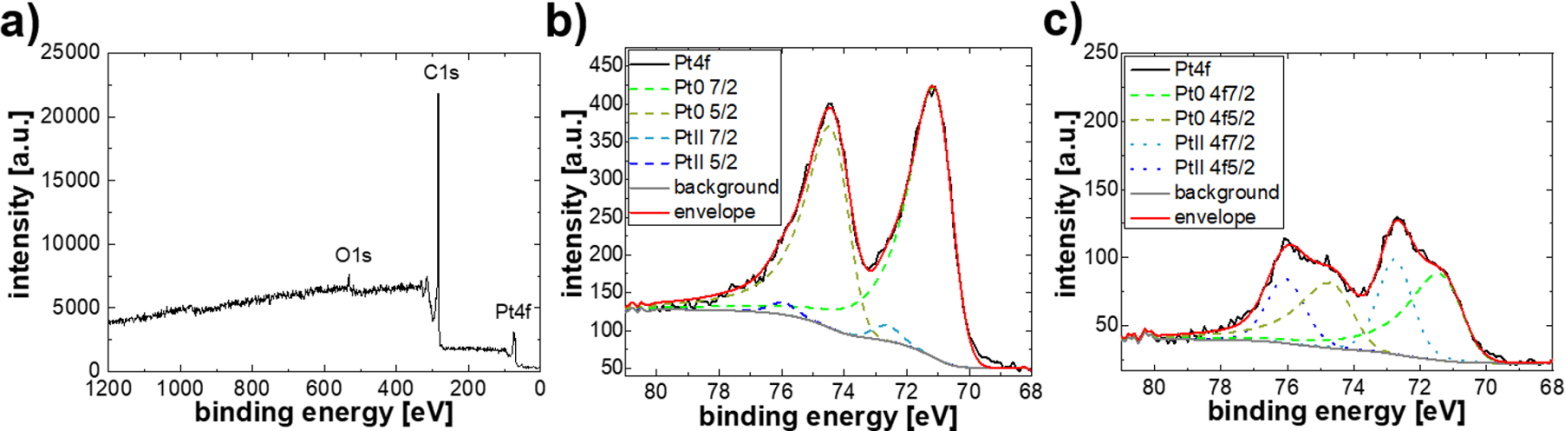
XPS a) survey and elemental scans of b) the slightly oxidized sample P7 and c) the highly oxidized sample P6. In the survey scan, no other signal besides those related to carbon, oxygen and platinum was detected. In b) and c) the oxidation degree is determined by fitting.

The relative platinum loading and degree of oxidation were determined by X-ray photoelectron spectroscopy (XPS, see Experimental section). In Figure 5, an XPS survey scan (Figure 5a) and Pt4f elemental scans (Figure 5b,c) related to the degree of oxidation of the Pt-NPs can be found. Other elemental signals besides carbon, oxygen, or platinum were not detected, which is expected, as the precursor Pt(acac)_2_ only consists of these elements and hydrogen. The Pt4f signal is divided into elemental Pt (Pt^0^) at ≈71 eV and oxidized Pt (Pt^II^), namely Pt(OH)_2_, at ≈72.7 eV.

Table 1 and Table 2 summarize weight percentages and degree of oxidation of the whole Pt/CNW layer (O content of Pt and C combined) and the degree of oxidation of only the Pt-NPs (Pt^0^/Pt^II^-ratio) as determined by XPS of samples obtained with different process pressures (Table 1) and carrier gas flow rates (Table 2). At a low substrate temperature of 350 °C, the platinum loading and degree of oxidation of both the whole Pt/CNW layer and the Pt-NPs alone largely depend on process pressure and carrier gas flow rate. The Pt loading was found to increase with decreasing pressure (Table 1: P3 vs P2 vs P4) and increasing carrier gas flow rate (Table 2: P6 vs P2 vs P7), which most likely results from decreased residence times (increased absolute gas velocity) of the educts. At high gas velocities, more Pt can reach the substrate, as at lower gas velocities Pt is already deposited in the silica glass tube leading from the oven to the deposition chamber. In contrast, low carrier gas flow rates and moderate to high pressures (small absolute gas velocities) resulted in substantial oxidation of the Pt-NPs in the layer, as can be seen when comparing sample P6 to P2. Remarkably, the degree of oxidation of the Pt-NPs can be controlled by varying the carrier gas flow rate at specific pressures without influencing the CNW morphology (as can be seen in Figure 4a). The increase in oxidation comes from an increased oxygen content in the plasma, which was verified by OES (see Supporting Information File 1, Table S2). In principle, a higher degree of oxidation of the catalyst could also be realized by introducing additional oxygen to the plasma via gas injection; however, oxygen is also known to influence the resulting CNW morphology [18–19]. At moderate Pt loading (7.6 wt %), the substrate temperature does not have any influence on Pt loading (not shown here).

**Table 1:** Weight percentages of Pt, C, and O of samples processed at different process pressures and a constant substrate temperature of 350 °C without the addition of hydrogen gas measured by XPS. The degree of oxidation of only the Pt-NPs (mainly Pt(OH)_2_) of individual samples is shown in parentheses under the Pt weight loading. The platinum loading and degree of oxidation of the whole Pt/CNW catalyst layers are dependent on the process pressure during synthesis.

Sample/Structure	Pressure [Pa]	Carrier gas flow rate [sccm]	Pt [wt%] (Pt^0^/Pt^II^ [%]/[%])	C [wt %]	O [wt %]

P3/CNWs	10.5	60	13.8 (97/3)	82.4	3.8
P2/CNWs	14.5	60	7.6 (98/2)	90.3	2.1
P4/nanoflakes	29	60	3.9 (60/40)	92.6	3.5

**Table 2:** Weight percentages of Pt, C, and O of samples processed at different carrier gas flow rates and a constant substrate temperature of 350 °C without the addition of hydrogen gas measured by XPS. The degree of oxidation of only the Pt-NPs (mainly Pt(OH)_2_) of individual samples is shown in parentheses under the platinum loading. The platinum loading and degree of oxidation of the whole Pt/CNW catalyst layers are dependent on the carrier gas flow rate during synthesis.

Sample/Structure	Pressure [Pa]	Carrier gas flow rate [sccm]	Pt [wt%] (Pt^0^/Pt^II^ [%]/[%])	C [wt %]	O [wt %]

P6/CNWs	14.5	30	7.6 (51/49)	88.4	4.0
P2/CNWs	14.5	60	7.6 (98/2)	90.3	2.1
P7/CNWs	14.5	93	15.0 (98/2)	82.0	3.0

The addition of hydrogen into the plasma reduces the degree of oxidation of the whole Pt/CNW layer (P10 vs P9 vs P8; Table 3), most likely due to a depletion of atomic oxygen, as determined by OES (Supporting Information File 1, Figure S3), and subsequent formation of water. The degree of oxidation of only the Pt-NPs does not change significantly with hydrogen addition (within an estimated margin of error of ±10%). At an elevated substrate temperature, similar effects occur (P10 vs P12 vs P11). With an increasing addition of hydrogen, the Pt loading of the catalyst also increases. This results from the etching effect of hydrogen on carbon structures, while Pt is not etched chemically.

An increase in substrate temperature reduces the platinum loading in the resulting hybrid material (P10 vs P12 and P9 vs P11; Table 3). As the precursor molecule provides much more carbon than platinum, the reduction in platinum loading likely results from a significant increase in the CNW growth and nucleation rate with substrate temperature (see Figure 4c) above the capability of the surrounding to provide sufficient Pt for embedding. Consequently, with careful adjustment of both the hydrogen concentration and the substrate temperature, the CNW morphology and the platinum loading can be tailored independently. For example, high wall densities and low loadings may be achieved at high pressures and high substrate temperatures, while low wall densities and high loadings can be achieved at low pressures with a moderate (0.5 sccm) addition of hydrogen.

**Table 3:** Weight percentages of Pt, C, and O of samples processed at varying substrate temperature and hydrogen addition, and at constant pressure (10.5 Pa) and overall carrier gas flow rate (argon and hydrogen gas; 60 sccm) measured by XPS. The degree of oxidation of only the Pt-NPs (mainly Pt(OH)_2_) of individual samples is shown in parentheses under the Pt weight loading. The platinum loading and degree of oxidation of the whole Pt/CNW catalyst layers are dependent on the substrate temperature and the addition of hydrogen during synthesis.

Sample/Structure	Temperature [°C]	Hydrogen addition [sccm]	Pt [wt%] (Pt^0^/Pt^II^ [%]/[%])	C [wt %]	O [wt %]

P10/CNWs	350	0	36.1 (86/14)	60.5	3.4
P9/CNWs	350	0.5	46.3 (89/11)	52.2	1.5
P8/a-C	350	1	68.1 (91/9)	31.5	0.4
P12/CNWs	500	0	21.5 (89/11)	75.5	3.0
P11/CNWs	500	0.5	28.0 (83/17)	69.4	2.6

#### Particle size

**Figure 6:**
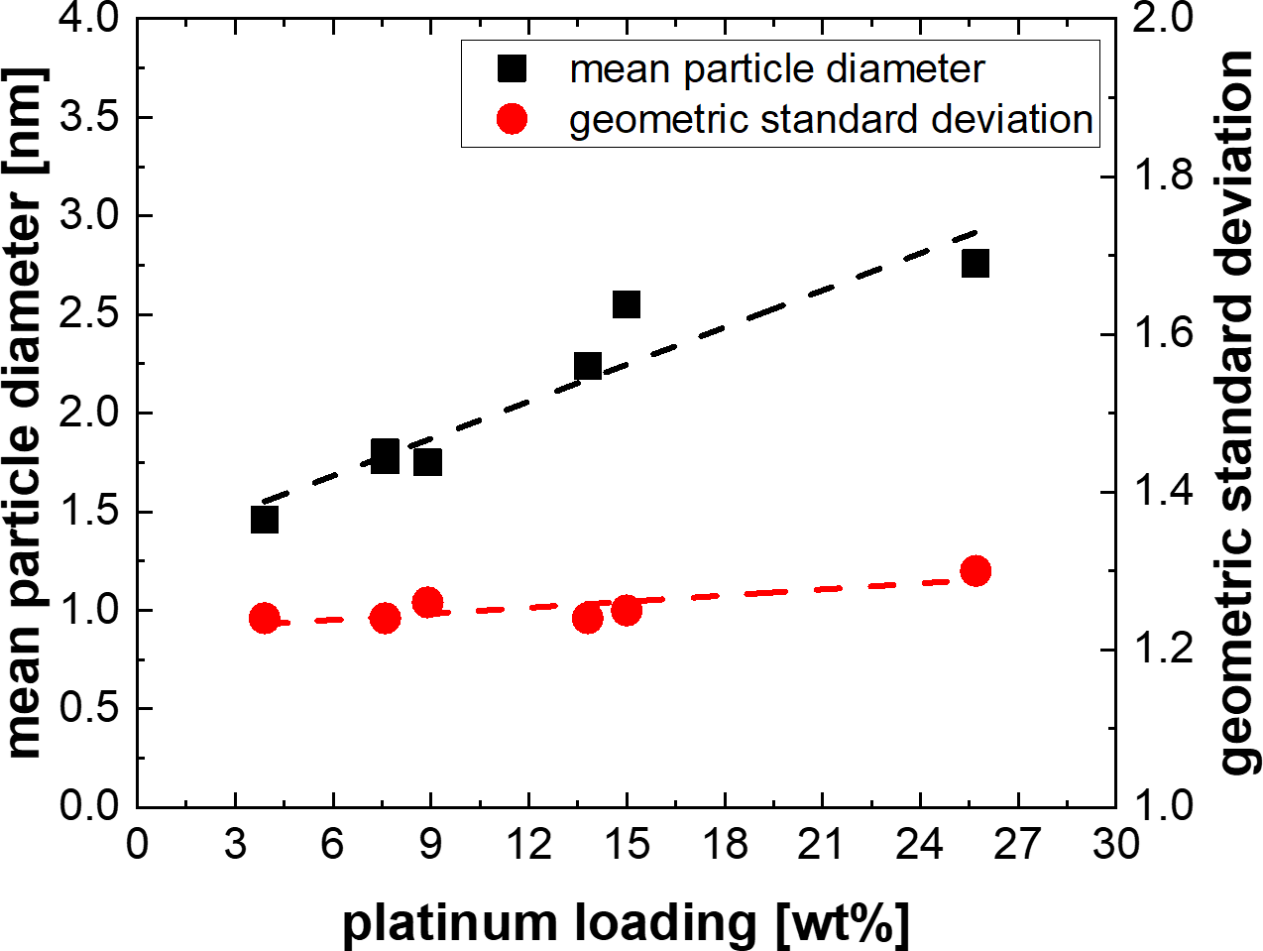
The dependence of mean particle diameter and geometric standard deviation on platinum loading of the catalyst layers for samples deposited at varying process parameters. Higher platinum loadings result in higher mean particle diameters, while the geometric standard deviation does not vary significantly.

Figure 6 shows the mean particle diameter (*x*_c_) and geometric standard deviation (σ_g_) over the platinum loading of Pt/CNW layers deposited under different process conditions. Since the platinum loading can be controlled by adjusting the absolute gas velocity of the precursor in the plasma (see section “Platinum loading and degree of oxidation”), the size *x*_c_ of the Pt-NP, which is strongly influenced by the platinum loading, can also be manipulated by tuning either the process pressure or carrier gas flow rate. In contrast, σ_g_ does not significantly vary between samples of different platinum loadings, resulting in narrow PSDs at any given platinum loading.

The high *x*_c_ values found at high gas velocities (high platinum loadings, see Table 1 and Table 2), indicate that the growth of the Pt-NPs does not occur in the gas phase but on the surface of the CNWs since nanoparticle growth in the gas phase should result in smaller particle sizes at high gas velocities (thus shorter residence time of the NP in the plasma), which is not the case here. Besides, the degree of precursor dissociation and hence the platinum concentration in the gas phase is expected to decrease with increasing gas velocities, as was previously demonstrated [17].

### Electrochemically active surface area and long-term stability

Even though the influence of the physicochemical properties of Pt/C on its electrochemical characteristics has been extensively studied in the literature [16,23], the unique synthesis method presented here is expected to influence both the electrochemically active surface area (ECSA), oxygen reduction reaction (ORR) mass activites and stability of the catalytically active Pt-NP, due to the aforementioned embedding. Therefore, Pt/CNW samples with different physical properties were studied concerning their ECSA, ORR mass activity and long-term stability, and the results were compared to those obtained with a commercially available Pt/C electrocatalyst.

**Figure 7:**
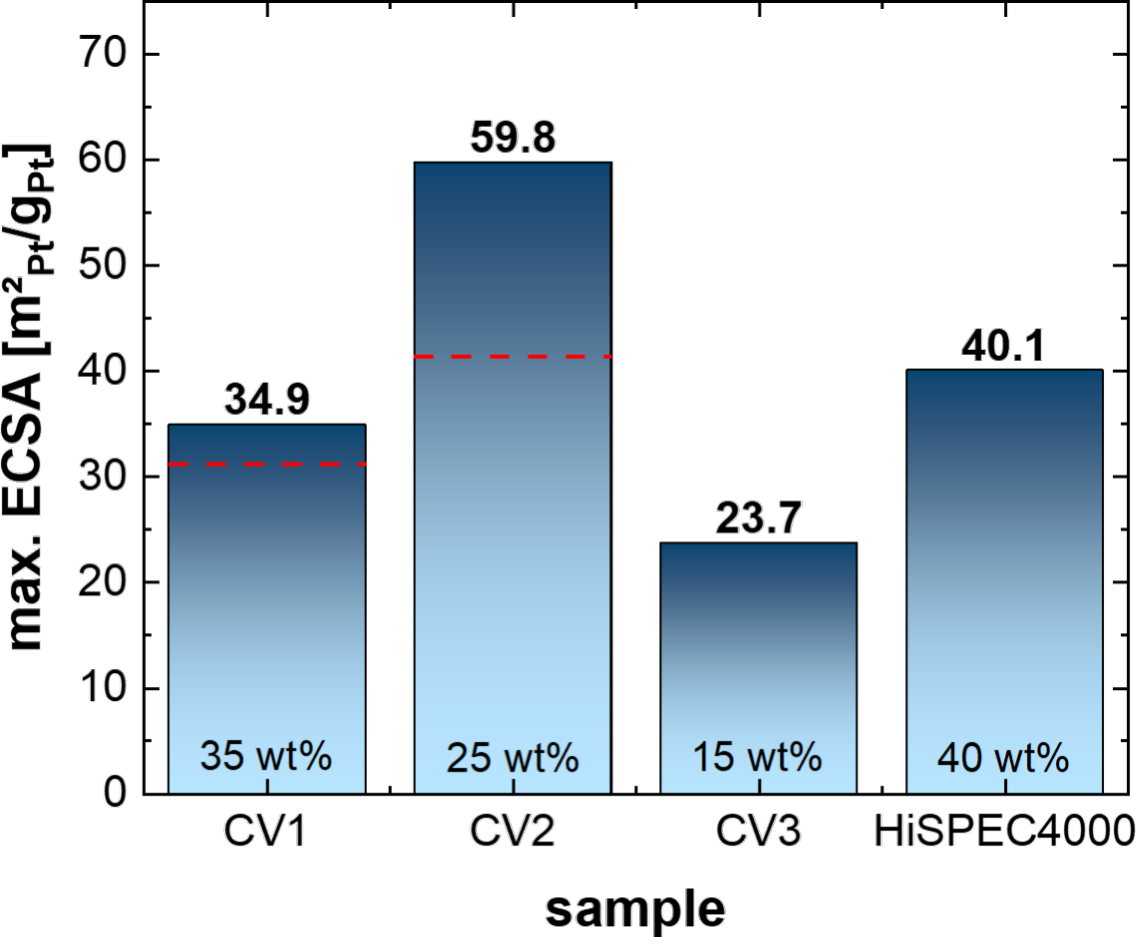
ECSA maximum (columns) and ECSA initial values (red, dashed line, if the ECSA_initial_ < ECSA_max._) for the Pt/CNW samples processed at different process parameters (CV1, CV2, and CV3) and a commercially available fuel cell catalyst (HiSPEC4000) measured by CV.

In Figure 7, the calculated ECSA (see Experimental section) of three samples (CV1, CV2, and CV3) are compared to a commercially available catalyst powder (HiSPEC4000). Relative platinum loadings (wt %) as determined by XPS are given in the columns in Figure 7, whilst the absolute platinum loadings were 39.0, 56.5, 17.5, and 20 µg/cm² for CV1, CV2, CV3 and HiSPEC4000, respectively. The calculated ECSA of all Pt/CNW samples is comparable to the ECSA of the standard commercial proton-exchange membrane fuel cell (PEMFC) catalyst, HiSPEC4000. The highest ECSA is found for sample CV2 and is approximately 1.5 times higher than that of the commercial catalyst. Note, however, that this difference most likely results from the much lower mean particle diameter of 2.76 nm (measured by TEM, see Supporting Information File 1, Figure S5) compared to 4.5 nm (measured by X-ray diffraction) of the commercial catalyst [33]. Corresponding PSDs of the three samples are given in Supporting Information File 1, Figure S4–6. The utilization (calculated ECSA divided by theoretical ECSA of smooth, round particles) is approximately 33%, 59%, 18% and 65% for CV1, CV2, CV3 and HiSPEC4000, respectively. The ECSA results show that not only the Pt particle size but also the morphology of the entire Pt/CNW structure plays a significant role – the highest ECSA and utilization are obtained for the Pt/CNW layer with the most dense structure (shorter CNWs), most likely resulting from improved contact between the Pt particles and the liquid electrolyte.

It is possible that the absolute ECSA values and utilization of CV1, CV2, and CV3 might be somewhat overestimated, due to the limitations described in the Experimental section. However, this does not affect the results for the significantly improved long-term stability of the Pt/CNW catalyst discussed in the following.

Traditional multistep synthesis methods for Pt/C catalysts use supports, which are partially graphitized for reaching the required high electrical conductivity and durability. Therefore, the support surface is characterized by a low density of binding sites for anchoring Pt-NP, on which the Pt-NPs can then easily diffuse. Hence, these supports often require additional functionalization steps for avoiding metal particle agglomeration, which reduces the electrical conductivity and durability of the support. In sharp contrast, the one-step synthesis of Pt/CNW presented herein results in a high density of anchoring sites for the co-deposited metal nanoparticles, which are partially embedded subsequently in the pristine carbon support with a high graphitization degree, and therefore, with high corrosion stability.

**Figure 8:**
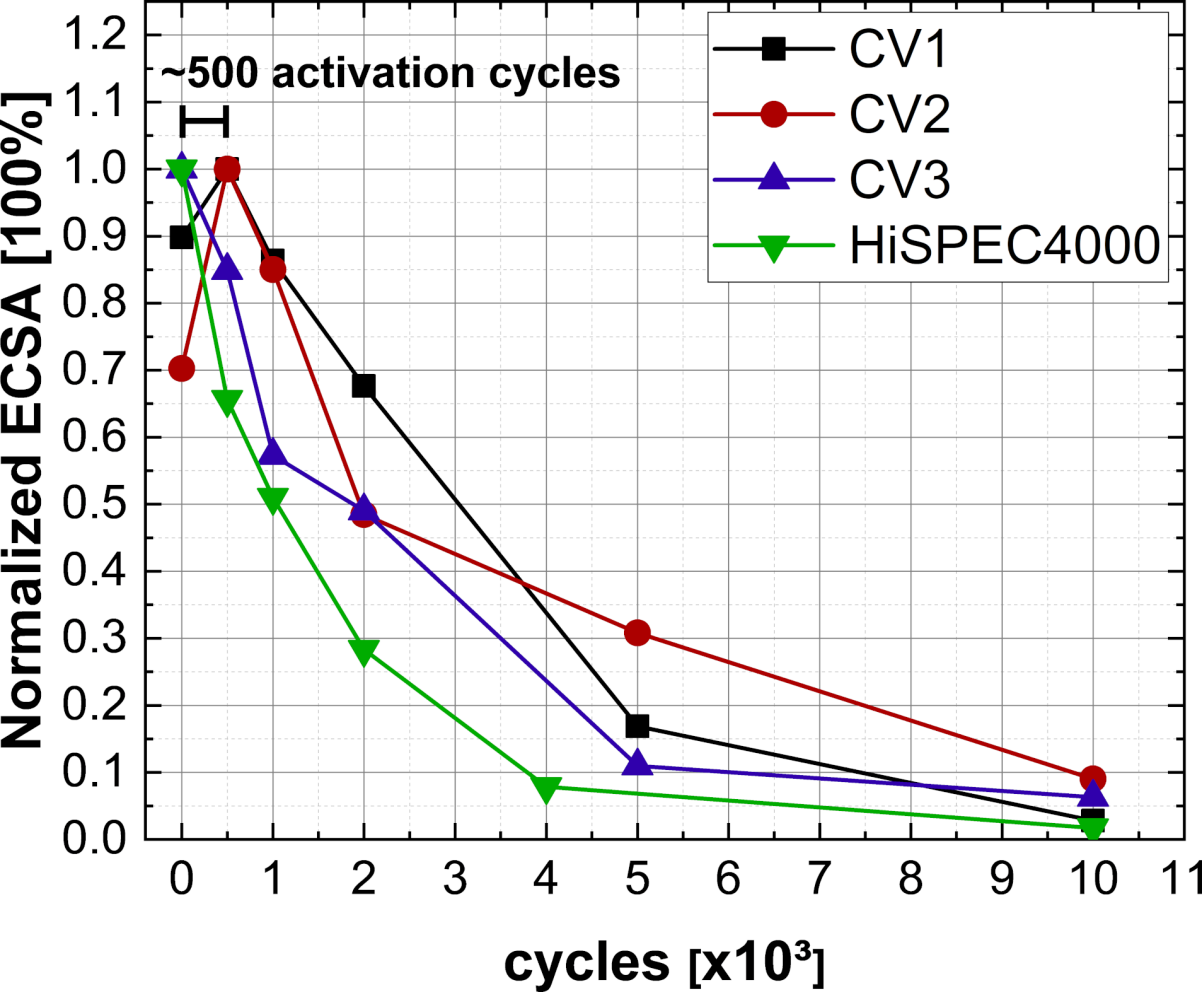
Normalized ECSA as a function of the AST cycles measured by CV. The superior long-term stability of the Pt/CNW samples CV1 and CV2, when compared to the commercially available HiSPEC4000, can be observed.

Consequently, embedding Pt-NP in the C-matrix not only results in narrow PSDs due to the suppression of particle growth by agglomeration but also in increased long-term stability as was observed for all Pt/CNW catalysts when compared to HiSPEC4000 (see Figure 8). The respective cyclic voltammograms of the samples mentioned in Figure 7 and Figure 8 are given in Supporting Information File 1, Figures S7–10. After 2000 accelerated stress test (AST) cycles, the most durable Pt/CNW layers still retain 50–70% of their maximum ECSA, while the commercial catalyst only has 30% left. Even after 5000 cycles, 20–30% of the maximum ECSA of the Pt/CNW layers are retained, at which point the commercial catalyst is already practically unusable. This increased long-term stability is surprising, as a significant percentage (at least >15% of the overall surface area at the highest mean particle diameter) of the PSD of our Pt/CNW catalyst layers are below or close to the threshold value for catalyst corrosion via Pt dissolution of 2 nm reported in the literature [16,34], which is not the case for HiSPEC4000 (mean particle diameter of 4.5 nm). After 10,000 cycles, no significant ECSA value could be measured for any of the catalysts.

Furthermore, during the first 500 cycles, the ECSA of samples CV1 and CV2 increases (see Figure 8), which likely results from the uncovering of partially carbon-coated Pt surfaces due to carbon corrosion during these cycles. These 500 cycles are seen as a positive feature of the Pt/CNW layers, as the ECSA increases from an already high, initial value. Additionally, we have measured ORR mass activities of the Pt/CNW samples relative to those of the commercial catalyst with a rotating disk electrode at 1600 rpm. Initially, after 0 AST cycles, none of the Pt/CNW samples are able to reach the activity of HiSPEC4000 with the best sample (CV2) reaching ≈33% of the mass activity of the commercial catalyst. However, as the AST continues, the Pt/CNW samples quickly overtake the commercial catalyst. After 500 AST, the best sample (CV1) shows two times the activity of the commercial catalyst, reaching almost five times after 2000 cycles. This can be explained by the aforementioned removal of carbon at the Pt-NPs surface and slower degradation of the Pt/CNW catalysts as observed in Figure 8.

However, the degradation mechanisms behind the ECSA loss on both Pt/CNW and HiSPEC4000 are quite similar, as seen from the exponential decay of ECSA in Figure 8. In the future, a broader electrochemical study of our Pt/CNW catalysts is required to further uncover the relations between particle embedding, ECSA, utilization, and ORR activity.

From the results of the ECSA, ORR, and accelerated stress test measurements, we find CV1 and CV2 to be the most promising electrocatalysts currently.

The presented synthesis method can be generalized for the deposition of many other metal/CNW hybrids by choosing the appropriate metal acetylacetonate. We have successfully used Al(acac)_3_ [17] and Pd(acac)_2_ as precursors, while others have demonstrated the use of Fe(acac)_3_ in a chemical vapor synthesis set-up [35]. This extends the application of the synthesis process described here far beyond the Pt/CNW system. In theory, the process would allow for functionalization of the carbon support or the deposition of technologically relevant nonprecious or alloyed metal catalysts to further improve long-term stability and cost-effectiveness [36]. Also, ongoing optimization of the synthesis method to realize a higher carbon graphitization, more favorable PSDs and better NP embedding is a promising way to increase long-term stability even further.

## Conclusion

A one-step, single-source approach for the deposition of Pt/CNW hybrid materials using plasma-enhanced chemical vapor deposition is presented. The wall density and height of the carbon matrix, as well as the platinum loading, degree of oxidation, and particle size distribution, can be precisely controlled by careful adjustment of the critical process parameters, such as working pressure, carrier gas flow rate, substrate temperature, and gas composition, which allows the formation of small Pt-NPs (*x*_c_ < 3 nm) with a narrow size distribution (σ_g_ < 1.3) even at high platinum loadings of more than 30 wt %. Compared to traditional synthesis methods, the simultaneous deposition of the Pt-NPs and the carbon matrix (CNWs) by using this novel approach yields Pt-NPs that are embedded in the C matrix and therefore show improved long-term stability compared to a commercial catalyst. Even though parts of the embedded Pt-NPs may not be in contact with the liquid electrolyte during CV, a sufficient ECSA and utilization were obtained.

The presented synthesis method is highly versatile, as a multitude of different metal acetylacetonates is commercially available, which facilitates the deposition of a variety of metal/CNW hybrids for optimization of performance and cost efficiency and use in applications other than electrocatalysis. In the current lab-scale non-optimized set-up, the yield of Pt/CNW catalyst is only approximately 1% that of the amount of precursor used, which is not cost-effective on an industrial scale. Nevertheless, we envision a roll-to-roll process in which the reactor size and gas feed are optimized, and the unused precursor (and Pt) is recycled and reintroduced into the process, which would greatly increase the yield.

## Experimental

The deposition process and methods are briefly described below. More details concerning the plasma system, synthesis, and characterization methods are given in a recent publication on the growth of Al/CNW hybrid materials from aluminum acetylacetonate [17].

### Synthesis

The deposition experiments are performed in an inductively coupled plasma-enhanced chemical vapor deposition system excited by 13.56 MHz radio frequency (RF). The system was constructed according to the guidelines of the gaseous electronics conference reference cell reactor [37] with a specially modified planar plasma antenna to increase plasma densities at low particle energies [38] and a slightly modified chamber geometry (see Supporting Information File 1, Figure S1).

Platinum acetylacetonate (98%, abcr GmbH) was placed inside an evaporator at the backside of the chamber, a silica glass tube was fitted to the exhaust of the evaporator that pointed towards the substrate holder, and the reactor chamber was evacuated to <5 × 10^−6^ mbar before the deposition process. The precursor was evaporated at a constant temperature of 128 °C, and argon flow was used to carry the precursor into the reaction zone of the plasma. The precursor flow rate was determined as constant at approimately 0.1 ± 0.01 sccm, which was found by weighing the amount of precursor before and after each synthesis using a high precision scale. The calculations were performed by using 
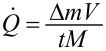
 where 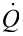 is the precursor gas flow rate, Δ*m* is the mass of evaporated precursor, *V* is the molar volume of an ideal gas (22.4 mol/L), *t* is overall process time (including preheating), and *M* is the molar mass of the precursor (393.302 g/mol). The material deposition was mainly done on 1 × 1 cm^2^ silicon substrates. In preparation for the cyclic voltammetry measurements, glassy carbon electrodes (GCEs; diameter 4 mm, Catalog No. 013338, ALS Co., Ltd.) were used as substrates instead.

The RF power was kept constant at 500 W (load power) and the processing time was 50 min if not stated otherwise. A pulsed bias voltage was applied to the substrate holder. The pulse shape was rectangular with a +30 V positive bias for 0.5 ms and −30 V negative bias for 5 ms to channel ionic species from the plasma onto the substrate. The chamber pressure, argon carrier gas flow rate, substrate temperature, and gas composition were adjusted, as stated in the Results and Discussion section. The plasma properties were monitored in situ by optical emission spectroscopy (see Supporting Information File 1).

### Characterization

The morphology of the resulting samples was investigated using a scanning electron microscope (SEM; Inspect F and Helios 600 NanoLab DualBeam, FEI Deutschland GmbH).

Raman spectroscopy was performed with green laser excitation (532 nm) in a Renishaw confocal inVia Raman microscope, and the spectra were evaluated using the method of Eckmann et al. [39] using standard data evaluation software (Origin).

A PHI Versaprobe II instrument was used to record XPS survey scans over a binding energy range of 0–1200 eV, and detailed elemental scans in the regions associated with C1s (≈284 eV), O1s (≈532 eV), N1s (≈400 eV), Pt4f (≈71 eV), Si2p (≈100 eV) and the Auger transition of C_KVV_ (≈1220 eV). All spectra were fitted with the software CASA XPS according to a method described elsewhere [17], while atomic percentages were determined with the software Multipak. The asymmetric line shape of elemental Pt [40] and the double peak splitting of 3.33 eV [41] was taken from bulk samples in the literature.

EDS (Oxford Instruments), in combination with an aberration-corrected transmission electron microscope (TEM; JEOL 2200FS), was used to analyze the chemical composition and to record high-resolution micrographs at a sub-nanometer level. An electron acceleration voltage of 200 kV was used. Particle size distributions were measured by using the semi-automatic mode of the software pebbles [42], assuming a spherical particle shape.

The electrochemical measurements were performed in a three-electrode electrochemical cell (glassy cell: double wall, temperature-controlled at 25 °C) filled with 0.1 M HClO_4_ electrolyte (AppliChem PanReac, 70%, for analysis, ACS, ISO). The working electrode is a GCE (diameter 4 mm, Catalog No. 013338, ALS Co., Ltd.) coated with the catalyst (by using the presented synthesis method). A Pt wire was used as the counter electrode, and Hg/HgSO_4_ (sat. K_2_SO_4_) from Radiometer Analytical with an electrode potential of 680 mV vs NHE (measured by us) was used as the reference electrode. The ECSA was calculated based on the H_ads_ peaks and a hydrogen monolayer adsorption charge of 210 mC/cm²(Pt). An accelerated stress test (AST) consists of 10,000 triangular potentiodynamic cycles between 0.4–1 V vs NHE with a scan rate of 1 V/s. ORR mass activities were measured at 1600 rpm in oxygen-saturated 0.1M HClO_4_ at 25 °C and determined at 0.9 V vs NHE.

When using GCEs as substrates for deposition and following characterization in CV, the deposited catalyst mass on these small substrates is in the range of 15–30 µg depending on Pt loading. These very low absolute catalyst masses are difficult to handle in inductively-coupled plasma mass spectrometry and prone to high measurement errors. Due to these limitations, the absolute platinum loading (in µg/cm²) used for the ECSA calculation was determined by measuring the absolute mass of a catalyst layer with a highly sensitive mass comparator (Sartorius C50) and multiplying it with the relative platinum loading (in weight percent) determined by XPS. For this, two GCEs were placed close to each other and coated with Pt/CNW in the same process. Afterward, both GCEs were compared to each other via SEM, Raman spectroscopy, and XPS. If the two GCEs were found to be almost identical (in terms of wall density, defect number/type, hybridization, and chemical composition), one of them was used in the CV (samples are designated as CV1–CV3). The other one was weighed with the mass comparator five times for reproducibility and afterward wiped clean of any remaining catalyst layer and weighed again, as a mass comparator can only measure a difference in mass with high accuracy (±1 µg), yielding the absolute catalyst mass. Some of the wiped off catalyst material was used for TEM measurements. Even though XPS is a surface-sensitive technique (to a depth of ≈5 nm), we are confident that this method gives a reasonable estimate of the absolute Pt loading used in the ECSA calculation, since the thickness of the well-separated CNWs is in this range (typically <5 nm).

## Supporting Information

File 1 Additional figures and tables.
