# Supplementary material for: One-step synthesis of carbon-supported electrocatalysts
Source: Beilstein J Nanotechnol. 2020 Sep 17;11:1419–31. doi: 10.3762/bjnano.11.126 (PMC7509379; doi:10.3762/bjnano.11.126)
Supplement: File 1 — Additional figures and tables. [file Beilstein_J_Nanotechnol-11-1419-s001.pdf]

BEILSTEIN JOURNAL OF NANOTECHNOLOGY

# Supporting Information

for

# One-step synthesis of carbon-supported electrocatalysts

Sebastian Tigges, Nicolas Wöhrl, Ivan Radev, Ulrich Hagemann, Markus Heidelmann, Thai Binh Nguyen, Stanislav Gorelkov, Stephan Schulz and Axel Lorke

*Beilstein J. Nanotechnol.* **2020,** *11,* 1419–1431. doi:10.3762/bjnano.11.126

# Additional figures and tables

License and Terms: This is a supporting information file under the terms of the Creative Commons Attribution License (https://creativecommons.org/licenses/by/4.0). Please note that the reuse, redistribution and reproduction in particular requires that the authors and source are credited.
The license is subject to the *Beilstein Journal of Nanotechnology* terms and conditions: (https://www.beilstein-journals.org/bjnano)

Schematic of the PE-CVD system:

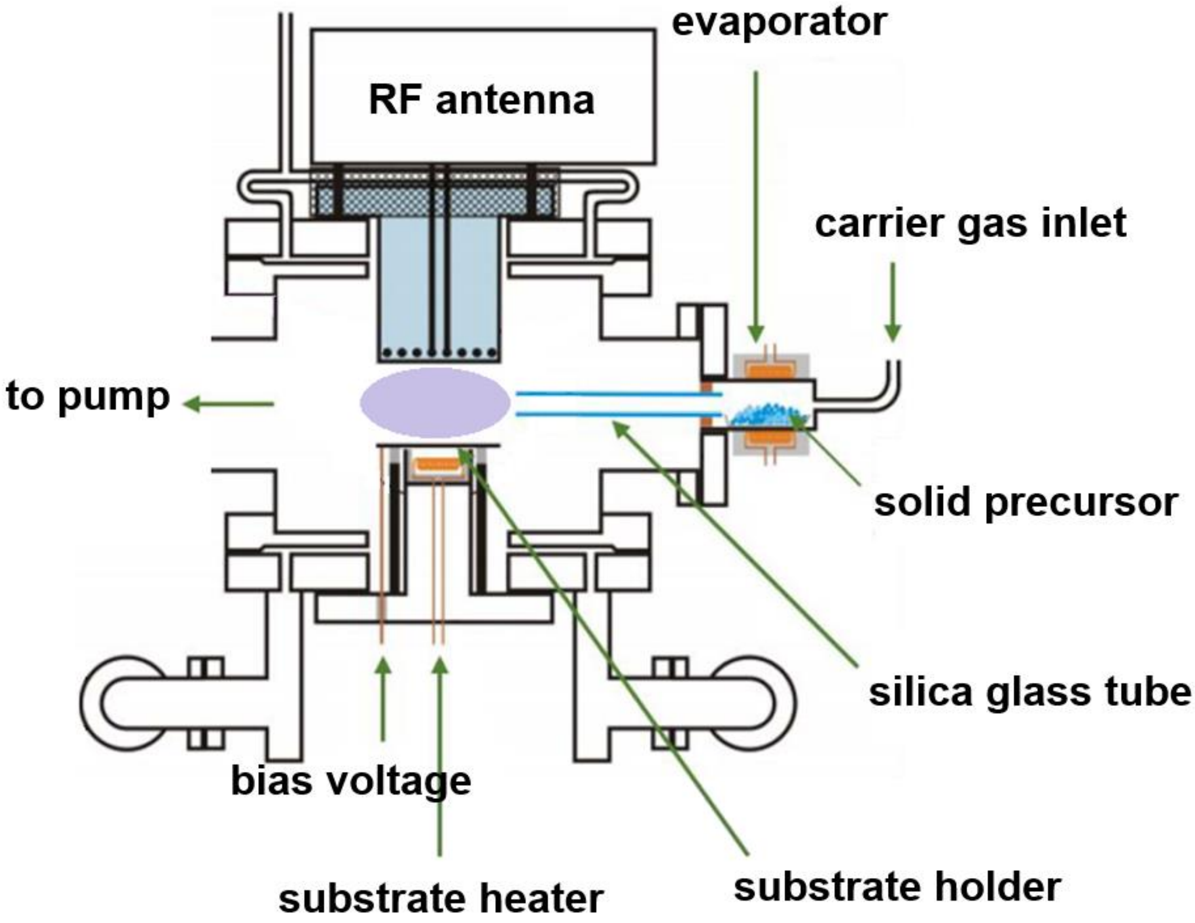


**Figure S1:** Schematic of the PE-CVD system used in this work.

*Insitu* OES:

Optical emission spectroscopy (OES) and actinometry were used to identify light-emitting species in plasma; therefore, the plasma's chemical composition. The optical characterization of the plasma during synthesis was performed with an iStar 340T Series CCD-camera fitted to a Shamrock 303i spectrograph. Spectra in this work are only qualitatively compared. Between samples, only trends in peak ratios are discussed.

Summary of all synthesis parameters used in this study:

**Table S1:** Summary of all synthesis parameters used for samples of this work. Only chamber pressure, carrier gas flow rate (Argon), substrate temperature, hydrogen (gas) addition, and distance to precursor inlet have been varied. Other process parameters, such as deposition time, bias voltage, and plasma power, were kept constant.

| **Sample** | **Pressure [Pa]** | **Carrier gas flow rate [sccm]** | **Substrate temperature [°C]** | **Hydrogen addition [sccm]** | **Position (distance to precursor inlet)** |
|---|---|---|---|---|---|
| **P1** | 14.5 | 60 | 425 | 0 | Si (medium) |
| **P2** | 14.5 | 60 | 350 | 0 | Si (medium) |
| **P3** | 10.5 | 60 | 350 | 0 | Si (medium) |
| **P4** | 29 | 60 | 350 | 0 | Si (medium) |
| **P5** | 14.5 | 60 | 500 | 0 | Si (medium) |
| **P6** | 14.5 | 30 | 350 | 0 | Si (medium) |
| **P7** | 14.5 | 93 | 350 | 0 | Si (medium) |
| **P8** | 10.5 | 59 | 350 | 1 | Si (close) |
| **P9** | 10.5 | 59 | 350 | 0.5 | Si (close) |
| **P10** | 10.5 | 60 | 350 | 0 | Si (close) |
| **P11** | 10.5 | 59 | 500 | 0.5 | Si (close) |
| **P12** | 10.5 | 60 | 500 | 0 | Si (close) |
| **CV1** | 10.5 | 59 | 500 | 0.5 | GCE (close) |
| **CV2** | 10.5 | 60 | 500 | 0 | GCE (close) |
| **CV3** | 10.5 | 60 | 500 | 0 | GCE (far) |

Raman spectroscopy of the deposited Pt/CNW layers:

Raman spectroscopy is a non-destructive characterization technique to investigate the electronic structure of any carbon allotrope and was performed on all samples. The defect-activated D and D' peak, which originate from the $A_{1g}$ breathing mode, in combination with the G peak, which results from the $E_{2g}$ phonon, can be used to gain insight into the defect density (amorphization and hybridization state) [S1] and type [S2]. The intensities/peak height of D, G, D' and 2D peak are later referred to as I(D), I(G), I(D'), and I(2D), respectively. A comprehensive review on Raman spectroscopy of carbon can be found elsewhere [S3].

Figure S2 a) shows typical Raman spectra of samples grown at different pressures and carrier gas flow rates. All of the samples produced at sufficiently high pressures and low carrier gas flow rates exhibit the typical spectrum observed for CNWs, resulting from their sharp, exposed edges [S4]. At low pressures and high carrier gas flow rates, the I(D') increases slightly, as observed in a previous study [S5]. The presumed reason for this is a transition of the defect type from boundary-like to monovacancy defect [S5], but the progression observed here is small. The amorphization stage of all the samples is 1, which indicates 100% $sp^2$-hybridization [S1]. This was later substantiated by measuring the D parameter of the $C_{KVV}$ line during XPS, where all of the samples show a near 100% $sp^2$-hybridization [S5]. In Figure S2 b) and c), the influence of substrate temperature and $H_2$ addition on the Raman spectrum of the deposited layers can be seen, respectively. While the substrate temperature does not change the course of the Raman spectrum substantially (Figure S2 b)) $H_2$ addition seems to increase the formation of monovacancy defects significantly, which can be observed with the increase in I(D') in Figure S2 c), until at a specific ratio of $Ar/H_2$ the layer becomes amorphous.

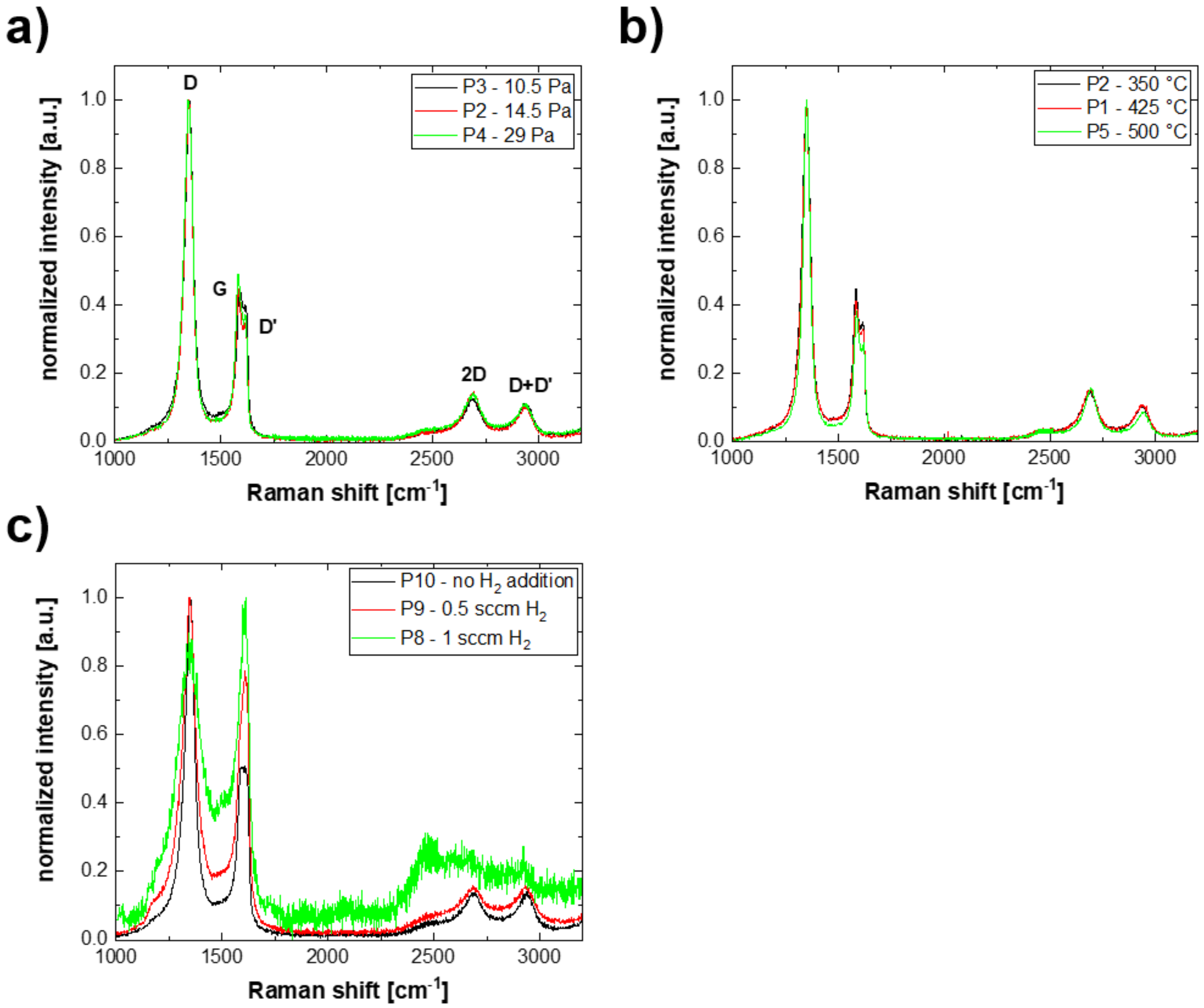


**Figure S2:** Raman spectra of samples processed at different process parameters. The influence of a) the pressure, b) the substrate temperature, and c) the addition of hydrogen during the process on the electronic structure can be observed. While pressure and substrate temperature have little impact on the electronic structure of the CNWs, the addition of hydrogen, even at low amounts, increases amorphization significantly.

Influence of hydrogen addition on other plasma species measured by OES:

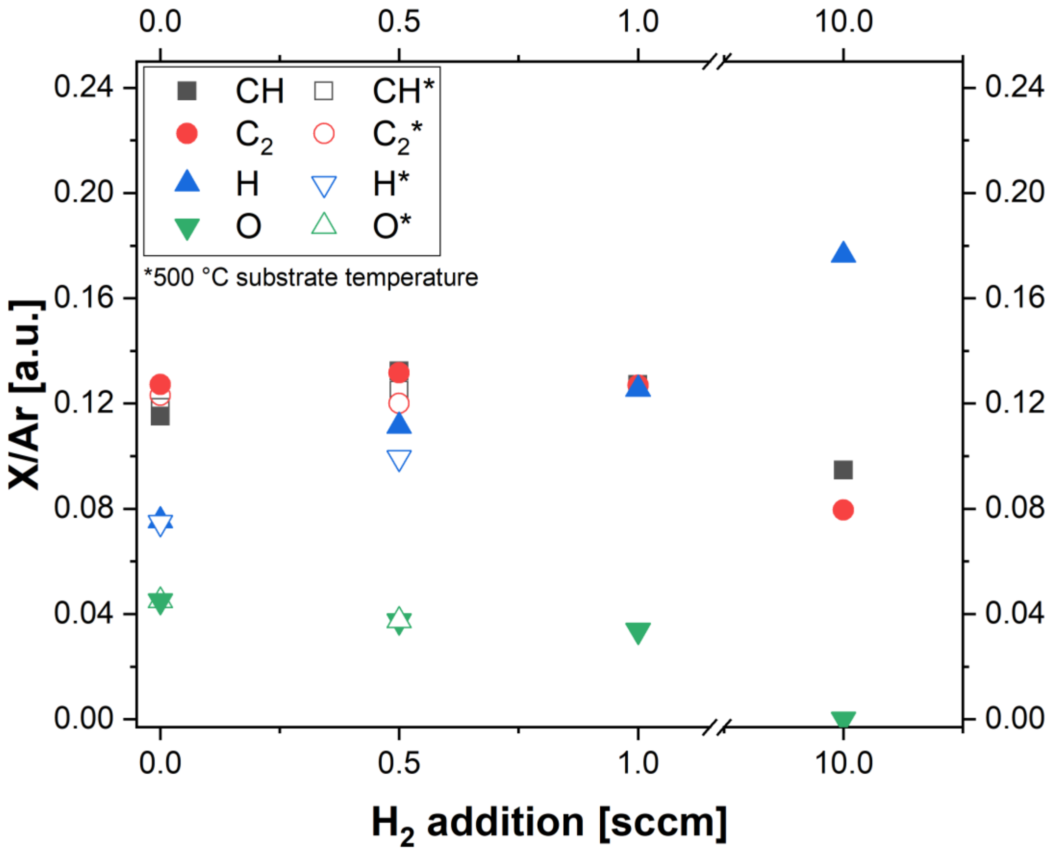


**Figure S3:** Influence of hydrogen addition during the process on the relative density of certain plasma species (X) normalized to the argon background gas measured by OES. A decrease in oxygen content with increasing hydrogen content in the plasma is observable.

Influence of pressure and carrier gas flow rate on the oxygen content in the plasma measured by OES:

**Table S2:** Oxygen content in the plasma normalized to the argon background gas for different pressures and carrier gas flow rates measured by OES. Increasing pressures and decreasing carrier gas flow rates result in increased oxygen content in the plasma.

| **Sample/Structure** | **Pressure [Pa]** | **Carrier gas flow rate [sccm]** | **Normalized O/Ar ratio [a.u.]** |
|---|---|---|---|
| P3/CNWs | 10.5 | 60 | 0.0741 |
| P2/CNWs | 14.5 | 60 | 0.07975 |
| P4/nanoflakes | 29 | 60 | 0.11033 |
| P6/CNWs | 14.5 | 30 | 0.22777 |
| P2/CNWs | 14.5 | 60 | 0.07975 |
| P7/CNWs | 14.5 | 93 | 0.05059 |

Particle size distributions of the samples used in cyclic voltammetry:

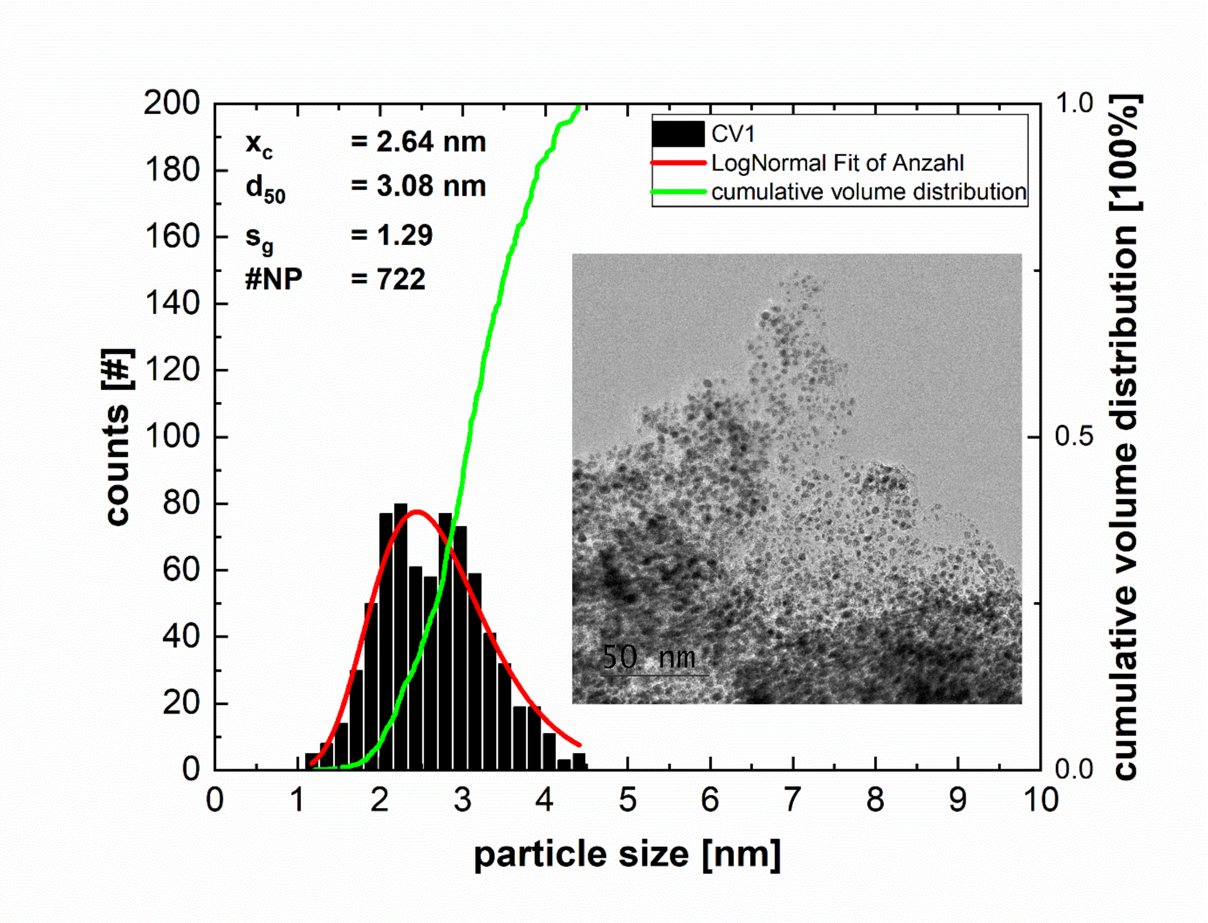


**Figure S4:** Histogram of the PSD of sample CV1 measured by TEM. The relative platinum loading of this sample determined by XPS was approx. 35 wt %. A narrow size distribution is evidenced by a low geometric standard deviation and the small difference between $x_c$ and $d_{50}$.

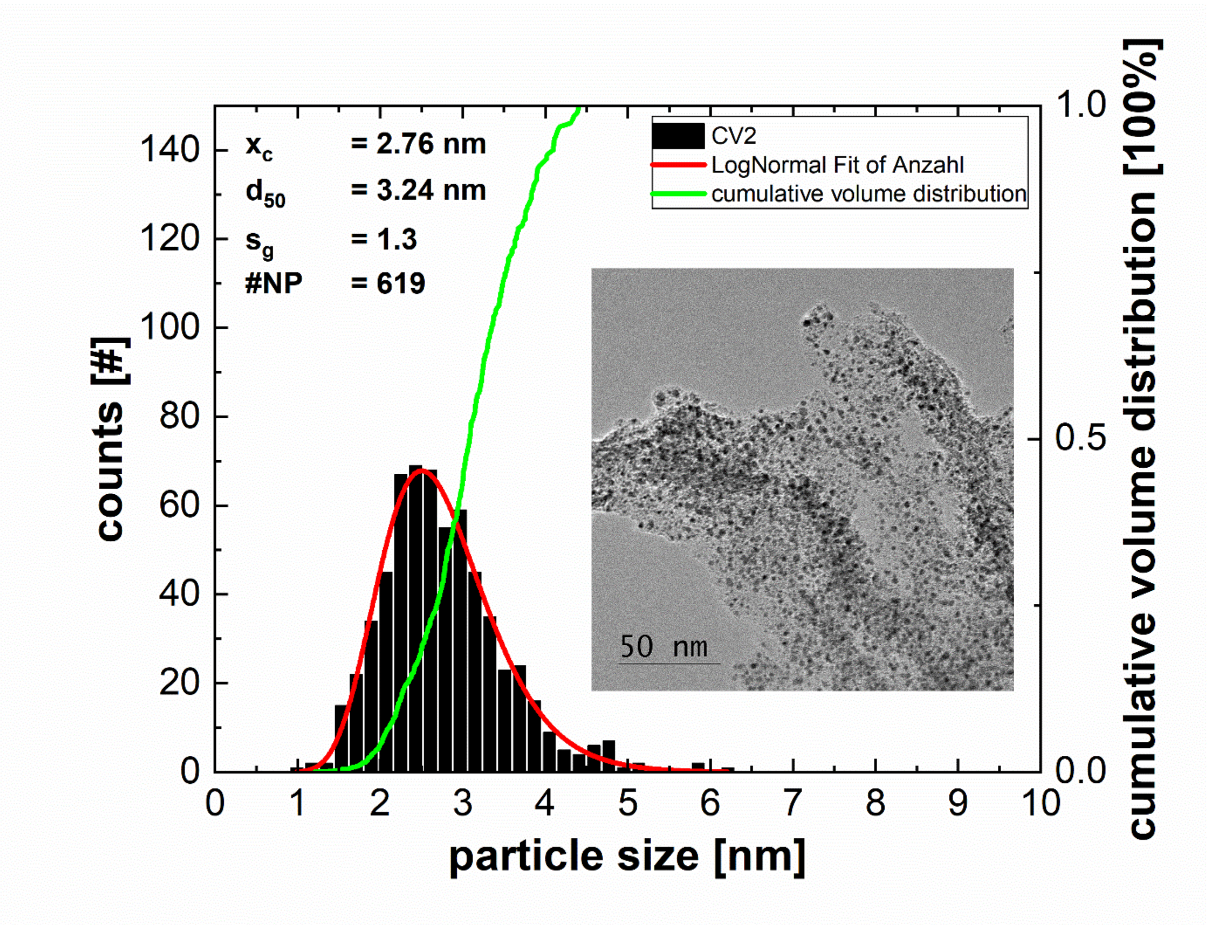


**Figure S5:** Histogram of the PSD of sample CV2 measured by TEM. The relative platinum loading of this sample determined by XPS was approx. 25 wt %. A narrow size distribution is evidenced by a low geometric standard deviation and the small difference between $x_c$ and $d_{50}$.

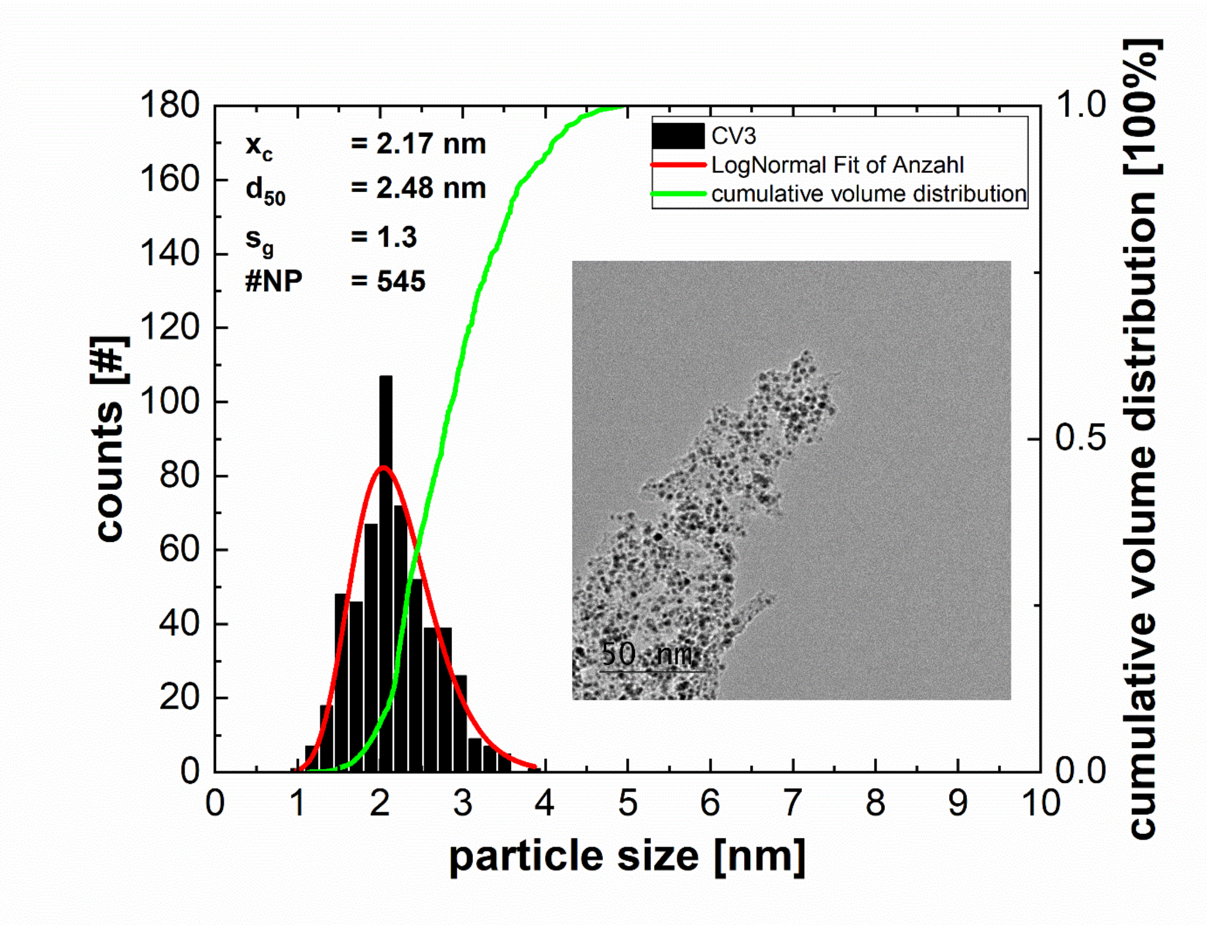


**Figure S6:** Histogram of the PSD of sample CV3 measured by TEM. The relative platinum loading of this sample determined by XPS was approx. 15 wt %. A narrow size distribution is evidenced by a low geometric standard deviation and the small difference between $x_c$ and $d_{50}$.

Cyclic voltammograms of the accelerated stress test measurements:

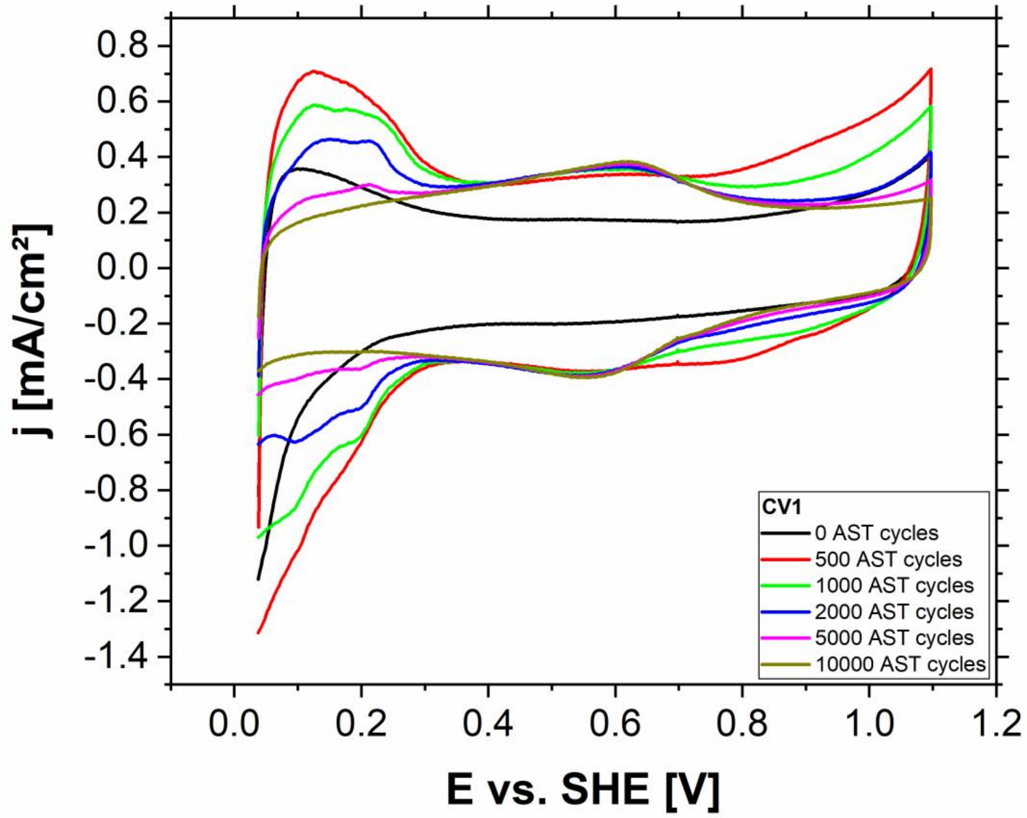


**Figure S7:** Cyclic voltammograms of the AST of sample CV1. The absolute Pt loading during the measurements was 39.0 µg/cm² (approx. 35 wt % relative platinum loading). The Pt peaks between 0 to 0.25 V are visible after an activation time of about 500 cycles. The ECSA (calculated from the Pt peaks) decreases with the number of AST cycles as expected.

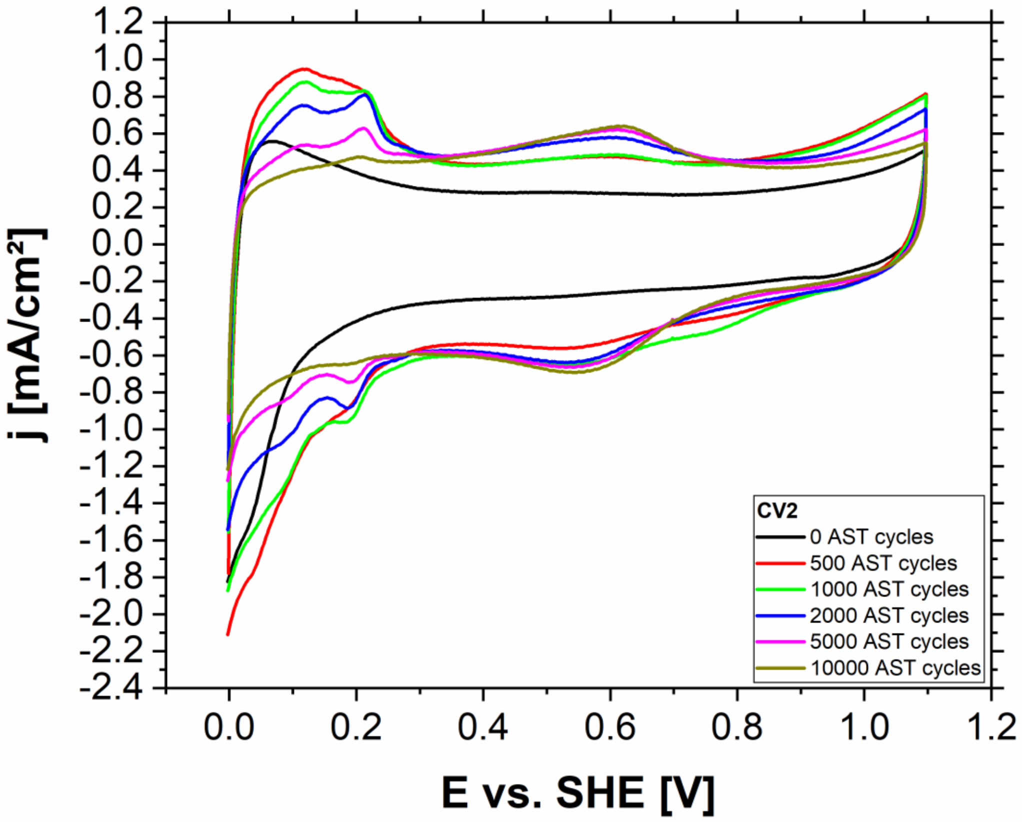


**Figure S8:** Cyclic voltammograms of the AST of sample CV2. The absolute Pt loading during the measurements was 56.5 µg/cm² (approx. 25 wt % relative platinum loading). The Pt peaks between 0 to 0.25 V are visible after an activation time of about 500 cycles. The ECSA (calculated from the Pt peaks) decreases with the number of AST cycles as expected.

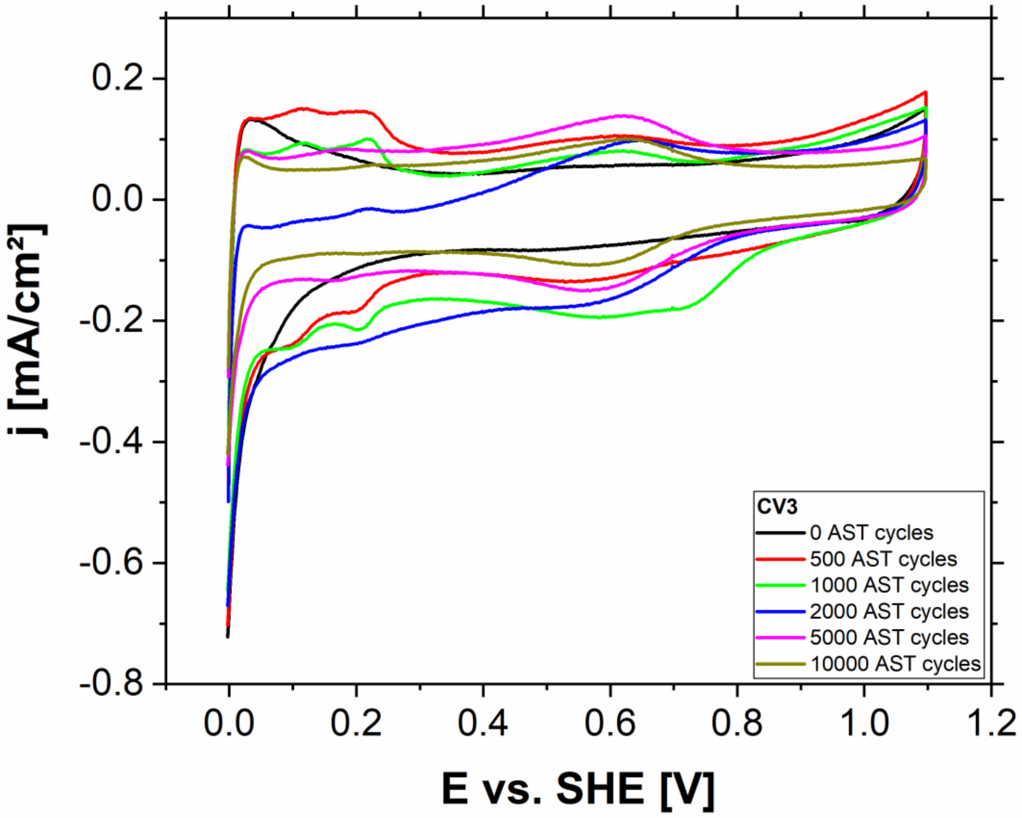


**Figure S9:** Cyclic voltammograms of the AST of sample CV3. The absolute Pt loading during the measurements was 17.5 ug/cm² (approx. 15 wt % relative platinum loading). The Pt peaks between 0 to 0.25 V are visible without activation. The ECSA (calculated from the Pt peaks) decreases with the number of AST cycles as expected.

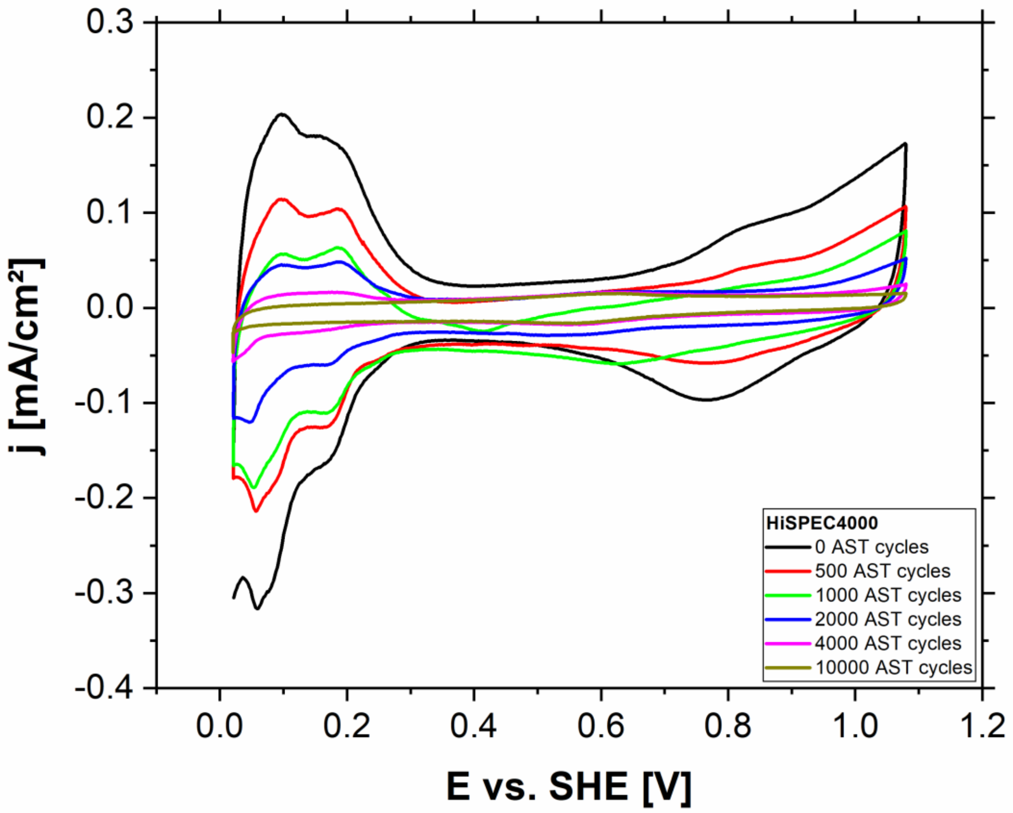


**Figure S10:** Cyclic voltammograms of the AST of sample HiSPEC4000. The absolute Pt loading during the measurements was 20 ug/cm² (approx. 40 wt % relative platinum loading). The Pt peaks between 0 to 0.25 V are visible without activation. The ECSA (calculated from the Pt peaks) decreases with the number of AST cycles as expected.

## References


(S1) Ferrari, A. C.; Robertson, J. *Phys. Rev. B* **2000**, *61* (20), 14095–14107. doi:10.1103/PhysRevB.61.14095

(S2) Eckmann, A.; Felten, A.; Mishchenko, A.; Britnell, L.; Krupke, R.; Novoselov, K. S.; Casiraghi, C. *Nano Letters* **2012**, *12* (8), 3925–3930. doi:10.1021/nl300901a

(S3) Ferrari, A. C.; Basko, D. M. *Nature Nanotechnology* **2013**, *8* (4), 235–246. doi:10.1038/nnano.2013.46

(S4) Ghosh, S.; Ganesan, K.; Polaki, S. R.; Ravindran, T. R.; Krishna, N. G.; Kamruddin, M.; Tyagi, A. K. *J. Raman Spectrosc.* **2014**, *45* (8), 642–649. doi:10.1002/jrs.4530

(S5) Tigges, S.; Woehrl, N.; Hagemann, U.; Ney, M.; Lorke, A. *J. Phys. D: Appl. Phys.* **2020**. doi:10.1088/1361-6463/ab6946

(S6) Kaciulis S. *Surface and Interface Analysis* **2012**, *44* (8), 1155–1161. doi:10.1002/sia.4892
